# Neural Correlates of Reward Processing: Impact of Individual Differences in Preference for Prosocial Interactions

**DOI:** 10.1002/brb3.70776

**Published:** 2025-09-08

**Authors:** Martina Vanova, Luke Aldridge‐Waddon, Ignazio Puzzo, Veena Kumari

**Affiliations:** ^1^ Department of Psychology, School of Life Sciences and the Environment Royal Holloway University of London Surrey UK; ^2^ Dementia Research Centre, Faculty of Brain Sciences, UCL Queen Square Institute of Neurology University College London London UK; ^3^ Centre For Cognitive and Clinical Neuroscience, College of Health, Medicine and Life Sciences Brunel University of London London UK; ^4^ Central and North West London NHS Foundation Trust London UK; ^5^ School of Psychology and Clinical Language Sciences University of Reading Reading UK; ^6^ Department of Psychology, College of Health, Medicine and Life Sciences Brunel University of London London UK

**Keywords:** fMRI, monetary and social incentive delay task, monetary reward, reward processing, prosociality, social reward, social reward questionnaire

## Abstract

**Introduction:**

There is an ongoing debate about the neural mechanisms and subjective preferences involved in the processing of social rewards compared to non‐social reward types.

**Methods:**

Using whole‐brain functional magnetic resonance imaging (fMRI), we examined brain activation patterns during the anticipation and consumption phases of monetary and social rewards (using the Monetary and Social Incentive Delay Task—MSIDT, featuring human avatars) and their associations with self‐reported social reward preferences measured by the Social Reward Questionnaire (SRQ) in 20 healthy right‐handed individuals.

**Results:**

In the anticipation phase, all reward types activated the dorsal striatum, middle cingulo‐insular (salience) network, inferior frontal gyrus (IFG), and supplementary motor areas. The consumption phase primarily engaged posterior cortical areas. Higher preference for prosocial interactions (as assessed by SRQ) was associated with increased right posterior cingulate activity during monetary reward anticipation and enhanced activity in the left striatum and salience network activation during social reward anticipation. In the consumption phase, higher prosociality was associated with stronger activation in frontal regions, the dorsal striatum, and the thalamus for monetary rewards and stronger putamen activity for social rewards.

**Conclusions:**

Individual differences in social reward preferences, particularly prosocial tendencies, are associated with distinct neural activations during reward processing. These findings have potential implications for understanding altered reward processing in clinical populations.

## Graphical abstract

• Reward anticipation activated the dorsal striatum and salience networks, while reward consumption engaged posterior cortical areas.• People with higher prosocial preferences show stronger left striatum activation during social rewards and right posterior cingulate activation during monetary rewards.

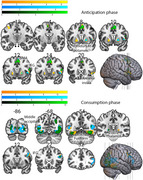

## 1 Introduction

Social rewards are features of social interaction associated with feelings of enjoyment and pleasure (Eisenberg et al., 2010; Foulkes et al., 2014). They often motivate social behavior, influencing how people seek and experience social contact (Gu et al., 2019; Rademacher et al., 2015). The neuropsychological mechanisms involved in social reward processing are less clear than those implicated in the processing of primary rewards (e.g., food or sex; Noori et al., 2016) and monetary rewards (Rademacher et al., 2010). Therefore, debate remains about associations between neuropsychological bases of social reward processing and corresponding subjective experiences of interpersonal interaction (Ait Oumeziane et al., 2017; Fareri & Delgado, 2014; Fulford et al., 2018). This may partly be linked to different definitions and characterizations of social reward experiences (Tamir & Hughes, 2018).

Reward processing comprises two separate temporal phases: anticipation and consumption. Reward anticipation (sometimes referred to as reward motivation or wanting) is characterized by the prospect of reward being encountered and approached (Oldham et al., 2018). Subsequently, reward consumption captures experiences of pleasure and satisfaction once the reward is obtained (Oldham et al., 2018). Current evidence from studies of social reward processing mechanisms (Bhanji and Delgado, 2014; Martins et al., 2021), as well as those investigating neuropsychological correlates of other reward processing types (e.g., monetary, primary) (Berridge et al., 2009; Jauhar et al., 2021; Lutz & Widmer, 2014) demonstrates that anticipation and consumption are associated with distinct behavioral and neuropsychological responses (e.g., Dillon et al., 2008; Smith et al., 2011) and thus should be conceptualized as discrete phases of reward processing.

Reward anticipation is associated with increased activation within the dorsal (caudate and putamen) and ventral (nucleus accumbens—NAcc) striatal regions (Knutson, Fong et al., 2001). Increased activations within the ventral striatum, the salience network (including the anterior insula and anterior cingulate cortex), the ventral tegmental area, the amygdala, and the thalamus, are described as neuropsychological features of reward anticipation across studies (Oldham et al., 2018; Wilson et al., 2018). The mesolimbic pathway is also implicated, with dopaminergic activity within and across areas involved in reward anticipation (Li et al., 2015). Comparatively, the brain areas involved in reward consumption are the orbitofrontal cortex (OFC) and ventromedial prefrontal cortex (vmPFC) (Levy and Glimcher, 2012), both of which play a role in how reward values are encoded and understood (Gläscher et al., 2009; Hiser and Koenigs, 2018). The neural dissociation of anticipation and consumption phases is supported by research (e.g., Oldham et al., 2018), which demonstrates that increased OFC, vmPFC, and posterior cingulate cortex activity is observed in the reward consumption phase only. As such, these findings demonstrate different brain regions associated with reward anticipation and consumption, positioning them as separable neuropsychological processes (Smith et al., 2011). Both phases are, of course, influenced by reward magnitude, probability, and expectedness (Diekhof et al., 2012; Liu et al., 2011), with heightened anticipatory and consummatory responses to rewards of larger magnitude.

There is continued debate whether the neuropsychological mechanisms involved in anticipation and consumption phases are similar across reward types—such as social versus non‐social rewards (Korb et al., 2020; Sailer et al., 2023). The same brain regions are implicated in the processing of both reward types (Gu et al., 2019; Sescousse et al., 2010), with some indication that monetary rewards might elicit more pronounced behavioral and neuropsychological responses than social rewards (Izuma et al., 2008; Lin et al., 2012; Rademacher et al., 2010; Spreckelmeyer et al., 2009). Many existing studies of social versus non‐social reward processing do not assess responses to different types of social reward. For example, Foulkes et al. (2014) posit that social reward can be delineated into six types (admiration, negative social potency, passivity, prosocial interactions, sexual relationships, and sociability). Thus, it may be important to account for these different reward types when characterizing the neuropsychological mechanisms of social reward processing. Moreover, many existing studies of social reward processing have often used a single social stimulus (e.g., a happy face) rather than stimuli that capture the multidimensionality of social reward experience (Matyjek et al., 2020). Indeed, studies of neuropsychological mechanisms implicated in the processing of specific forms of social reward, such as admiration, demonstrate that social media “Likes” are associated with reward network responses during reward consumption, shown as increased bilateral NAcc activation while looking at photos with more ‘Likes’ than photos with less ‘Likes’ (Sherman et al., 2016). Moreover, features of prosociality, like altruistic giving (Cutler and Campbell‐Meiklejohn, 2019), including others (Kawamichi et al., 2019; van der Meulen et al., 2016), and fairness (Tabibnia et al., 2008), are linked to increased activation within vmPFC, amPFC, precuneus, subgenual anterior cingulate cortex, and OFC regions.

There is increasing evidence that the processing of different types of social rewards has an influence on interpersonal behavior in psychopathology, including reduced prosocial behavior in antisocial personality disorder, social withdrawal in depression, and adjusted reciprocity in autism spectrum conditions (Aldridge‐Waddon et al., 2022, 2020; Chen et al., 2022; Foulkes et al., 2015; Raihani et al., 2021). Thus, it is important to establish whether these different social reward types are also associated with different neuropsychological mechanisms. Doing so might provide a more comprehensive understanding of the neuropsychological processes involved in social reward anticipation and consumption and individual differences therein. It could inform the development of targeted interventions that address specific reward‐processing responses rather than treating social reward as a unitary construct.

The present study aimed to identify differences in neuropsychological responses during the anticipation and consumption of non‐social and social rewards. Specifically, we aimed to identify patterns of brain activation for monetary and social reward types and examine their association with self‐reported subjective values of different types of social reward in real‐life scenarios. We examined how individual differences in social reward preference might relate to neural responses during reward anticipation and consumption. To achieve our aims, we used a Monetary and Social Incentive Delay Task (MSIDT) that involved complex scenarios and featured human avatars in all reward types during functional magnetic resonance imaging (fMRI). We tentatively hypothesized that anticipation and consumption of both social and non‐social rewards would be associated with increased activation in the brain areas implicated in reward processing, such as dorsal and ventral striatal regions (including NAcc), OFC, and vmPFC. Secondly, we expected positive associations between a (higher) preference for social rewards and brain activations during anticipation and/or consumption of social, relative to neutral and monetary, rewards.

## 2 Methods

### 2.1 Participants

Twenty healthy right‐handed adults (7 males, 13 females; age M = 24.35, SD = 5.25) were recruited from the Brunel University of London network. All participants reported no history of mental illness or instrumental violence. Participants were requested to refrain from using alcohol or any drugs (except for usual caffeine consumption) on the day of their study participation. The Research Ethics Committee of Brunel University of London approved the study. All participants provided written informed consent before participating and were compensated £20 for their time.

### 2.2 Materials

#### 2.2.1 Self‐report Measure of Social Reward Preference

All participants completed the Social Reward Questionnaire (SRQ) (Foulkes et al., 2014). The questionnaire consists of 23 items divided into six subscales: (a) Admiration, (b) Negative Social Potency, (c) Passivity, (d) Prosocial Interactions, (e) Sexual Reward, and (f) Sociability. The answers are collected on a 7‐point Likert scale (strongly disagree = 1, strongly agree = 7), with higher scores indicating social experiences as more rewarding. The SRQ is a widely accepted tool for assessing individual differences in social reward processing in normative and clinical populations (Chen et al., 2022; Foulkes et al., 2015; Raihani et al., 2021). It has excellent psychometric properties (Cronbach's Alpha (α): mean = 0.82, SD = 0.04, range = 0.77–0.87; test‐retest (*r*): mean = 0.80, SD = 0.06), with good construct and content validity (Foulkes et al., 2014) and a verified six‐factor structure (Smeijers et al., 2022).

#### 2.2.2 fMRI: Paradigm and Procedure

**FIGURE 1:**
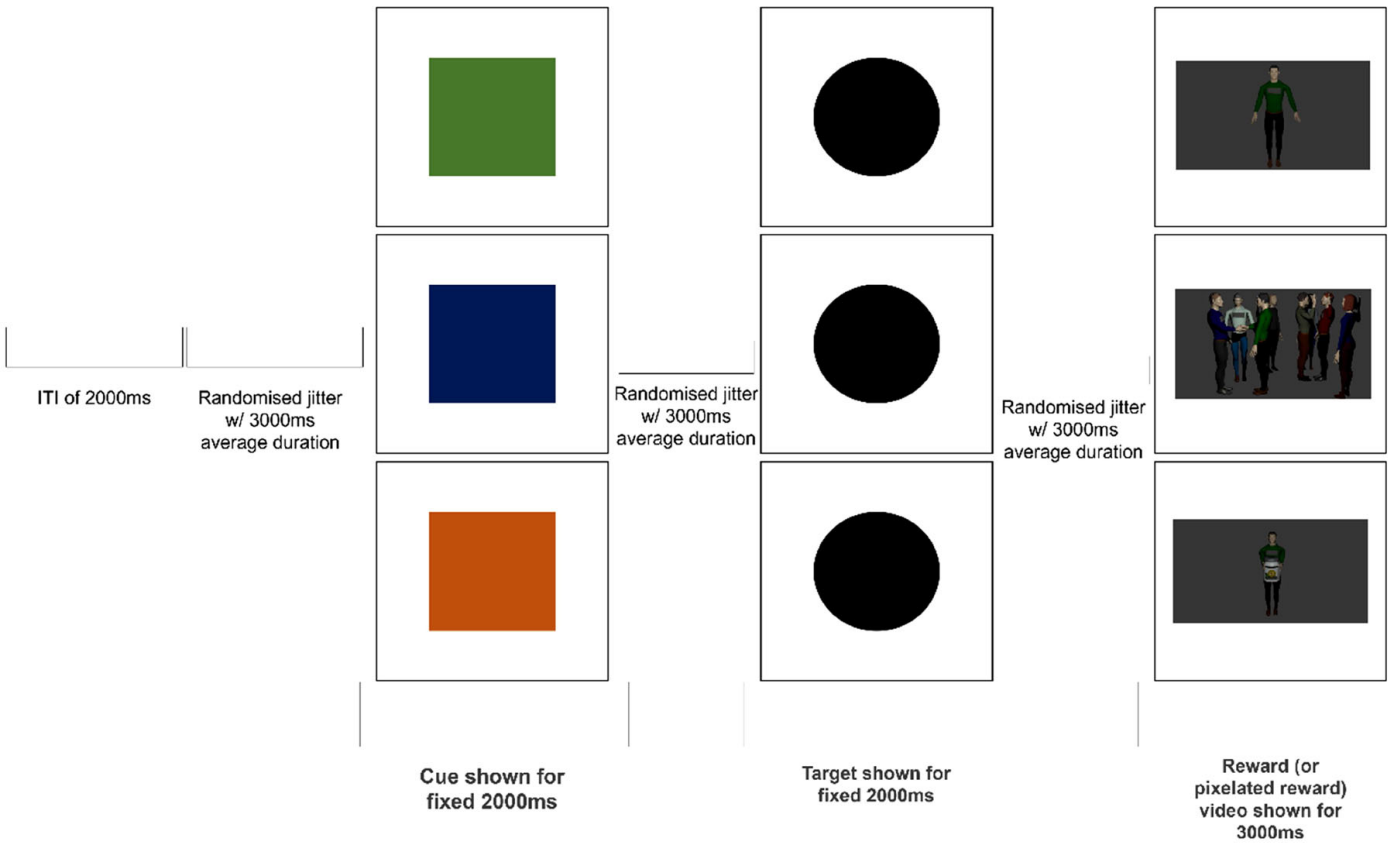
MSIDT trial featuring cues for monetary (orange), social (blue), and neutral (green) reward conditions and the corresponding reward videos.

The MSIDT used during fMRI was designed to assess sensitivity to monetary versus social rewards (based on Aldridge‐Waddon et al., 2022). Before the fMRI started, participants were asked to select the avatar to play as. During the fMRI, participants were presented with a series of anticipatory cues (orange square—monetary reward, blue square—social reward, green square—neutral reward) and then with a target (black circle) that they had to respond to as fast as possible by pressing a button (Figure 1). A four‐button MRI‐compatible response box (Lumitouch, PhotonControl Inc., Baxter, Canada) was used to record responses. If a participant responded faster than the average RT in the practice session, they would earn one of the hypothetical rewards: monetary (avatar holding a coin jar), social (avatar engaging in a group activity, e.g., talking with others, teasing others in a group, receiving applause, etc.), or neutral (avatar standing alone). Each trial with a fast response was marked as a reward won, representing task accuracy. For trials with no reward won, a pixelated image appeared instead of the reward video. There were 90 trials (30 per reward condition) in three blocks, with the blocks separated by a 20‐second blank screen. Each trial (Figure 1) lasted approximately 18 s, consisting of an inter‐trial interval (ITI) (duration 2000 ms), 1000–5000 ms jitter (average duration 3000 ms), a reward cue (2000 ms), an ISI (average duration 3000 ms), a target (2000 ms), an ISI (average duration 3000 ms), and a reward video (3000 ms). The task took an average of 27 minutes to complete.

### 2.3 fMRI Data Acquisition

The functional images were acquired using the pulse sequence. TR = 2000 ms, TE = 30.6 ms, 50 interleaved slices, voxel size = 2×2×3 mm, flip angle = 78°, field of view = 192 mm, base resolution = 96, 96×96 matrix. Time correction was based on the middle slice, and the realignment reference volume was the first volume. High‐resolution T1‐weighted images were acquired with the following settings: TR = 2300 ms, TE = 2.9 ms, 192 images of 1×1×1 mm voxel size, flip angle = 9°, field of view = 256 mm, base resolution = 256, matrix 256×256.

### 2.4 Statistical Analyses

Behavioral data analyses were performed using IBM SPSS Statistics, V26.0 (IBM Corp., 2019), with *p* < .05. The percentage of trials where a reward was won (out of the total number of possible trials; 30 per reward type) and RTs for target stimuli were analyzed using a 3 (reward type) × 2 (sex) Analysis of Variance (ANOVA) with reward type (monetary, social, neutral) as a within‐subject factor and sex (males, females) as the between‐subject factor. The Greenhouse–Geisser correction was applied where Mauchly's Test indicated a significant sphericity violation. Pearson's correlation coefficients (*r*) were used to examine associations between the SRQ scores (Admiration, Prosocial Interactions, Sexual Reward, and Sociability) and MSIDT performance (i.e., % of trials with a reward won). MSIDT variables that were significantly associated with two or more SRQ subscales were analyzed further using the linear regression ‘Stepwise’ method. This method determines the final model based on a process of selecting/eliminating predictors one at a time depending on the outcome of the t‐tests for the slope parameters (i.e., partial F‐tests) and the amount of shared and unique variance explained by these predictors using the commonality analysis.

MRI data were pre‐processed and analyzed using the SPM12 toolbox (Friston et al., 2007) for MATLAB (2020) and MRIcroGL (Rorden and Brett, 2000) for graphic visualization. First, the anterior commissure was set as an origin for the structural and all functional images. Subsequently, functional images were realigned and co‐registered with the corresponding structural images. The resulting images were normalized to the Montreal Neurological Institute coordinate system (MNI) space with 2×2×2 mm voxel resolution for functional images and forward deformation fields. The transformation parameters were derived from the segmentation of structural images. The normalized images were smoothed with a full width at half‐maximum (FWHM) Gaussian smoothing kernel of 6 mm.

We conducted a two‐level analysis of the pre‐processed images. At the first level, a random‐effect analysis of participant‐specific contrast activations (i.e., three reward types at the anticipation phase and consumption phase compared to the implicit baseline‐resting condition—monetary, social, and neutral rewards each over baseline and one another—combinations of the three stimuli‐types. At the second level, we identified task‐related neural activations using one‐sample t‐tests across the sample (height threshold 𝑝 < 0.0001 uncorrected; FWE correction for multiple comparisons at the cluster level 𝑝 < .05) (Supplementary Tables 1 and 2, anticipation and consumption phases, respectively). We examined the relationships of individual differences in social reward preference, as indexed by the Admiration, Prosocial Interactions, Sexual Reward, and Sociability subscales of the SRQ, with MSIDT‐related neural activations (for each contrast) across the whole brain using a regression model within SPM12 (height threshold 𝑝 < 0.001 uncorrected; FWE corrected at cluster level 𝑝 < 0.05) (Supplementary Tables 3 and 4, anticipation and consumption phases, respectively).

## 3 Results

### 3.1 MSIDT Performance and Subjective Social Reward Preference

Descriptive statistics for the MSIDT and the SRQ are presented in Table 1. One participant was excluded due to incomplete data on the SRQ. No significant effect of Sex or Reward Type was found in MSIDT performance (all *p* > 0.05).

**TABLE 1:** Descriptive statistics for the MSIDT performance and the SRQ scores.

MSIDT (*n* = 20)	Mean	SD	Minimum	Maximum
**Monetary Accuracy** [%]	53.83	22.35	16.67	90.00
**Social Accuracy** [%]	49.67	25.31	10.00	93.33
**Neutral Accuracy** [%]	49.50	22.22	13.33	86.67
SRQ subscales (*n* = 19)	**Mean**	**SD**	**Minimum**	**Maximum**
**SRQ Admiration**	22.68	5.75	4	28
**SRQ Negative Social Potency**	7.84	3.13	5	15
**SRQ Passivity**	9.37	4.35	4	17
**SRQ Prosocial Interactions**	31.84	3.53	21	35
**SRQ Sexual Reward**	13.79	4.95	3	21
**SRQ Sociability**	14.58	3.79	4	19

*Note*: RT—reaction time.

### 3.2 Associations Between MSIDT Performance and Individual Differences in Social Reward Preferences

When significant correlations between the MSIDT accuracy (rewards won) and SRQ subscales (Table 2) were inputted into the regression model, only Prosocial Interactions was accepted as a significant predictor of monetary rewards won, accounting for 35% of the variance [F(1, 17) = 9.328, *p* = 0.007, *R^2^* = 0.354]. In addition, Admiration scores positively correlated with social rewards won (*r* = 0.498, *p* = 0.030).

**TABLE 2:** Pearson correlations of MSIDT performance with the SRQ scores (*n* = 19).

	Accuracy [%]		
	Monetary	Social	Neutral
**SRQ Admiration**	0.595**	0.498*	0.165
	0.007	0.030	0.499
**SRQ Negative Social Potency**	0.088	0.197	−0.052
	0.720	0.418	0.834
**SRQ Passivity**	0.322	0.089	0.260
	0.178	0.718	0.281
**SRQ Prosocial Interactions**	0.475*	0.320	0.310
	0.040	0.182	0.197
**SRQ Sexual Reward**	0.154	0.223	−0.154
	0.529	0.359	0.528
**SRQ Sociability**	0.372	0.292	0.145
	0.117	0.224	0.553

*Note*: ** Significant at 0.01 level (two‐tailed); * Significant at the 0.05 level (two‐tailed).

### 3.3 Post‐hoc Power Calculation

For a two‐tailed test, with 20 participants and the strongest encountered effect (Pearson's correlation *r* = 595, with α = 0.05 as Type I error probability), we obtained 93% power.

## 4 fMRI

### 4.1 Task‐related Activations

**FIGURE 2:**
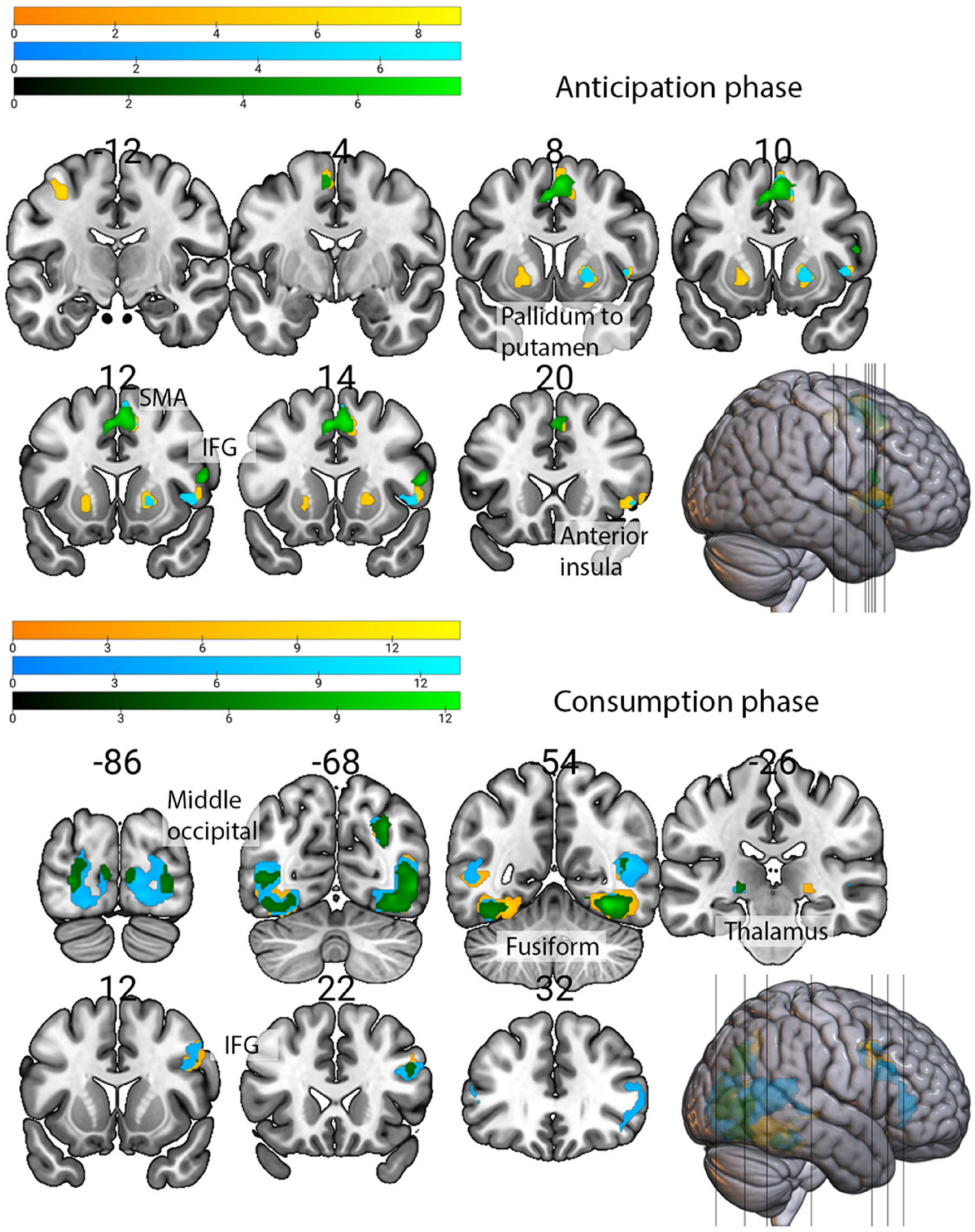
Task‐related activations for **(a)** Monetary (yellow), **(b)** Social (blue), and *(c)* Neutral (green) rewards contrasts over baseline during the anticipation and consumption phases (coronal view, y plane) (*N* = 20).

In the anticipation phase (Figure 2), monetary reward cues activated mainly the pallidum to putamen bilaterally, the right anterior insula, and the pars opercularis of the IFG. Social reward cues activated these areas on the right side. Neutral cues did not activate any striatal areas and activated only the pars opercularis of the right IFG and left supplementary motor area. All contrasts were significant when compared to the baseline, but direct comparisons of different reward anticipations did not yield any significant differences (Supplementary Table 1).

In the consumption phase, primarily posterior cortical regions were activated, mainly the right fusiform gyrus, right temporal, and inferior frontal regions, in all conditions compared to baseline. The left thalamus was activated during the consumption of monetary and social rewards (Figure 2). Monetary rewards won activated the left fusiform gyrus more strongly compared to social rewards won, whereas social rewards compared to monetary rewards activated more strongly in the occipital areas bilaterally and the right fusiform gyrus and temporal areas. Consumption of neutral rewards activations mostly overlapped with monetary and social rewards activations with additional activity in the calcarine and lingual gyri bilaterally (Supplementary Table 2).

### 4.2 Relationship Between Task‐related Activations and Social Reward Preference

**FIGURE 3:**
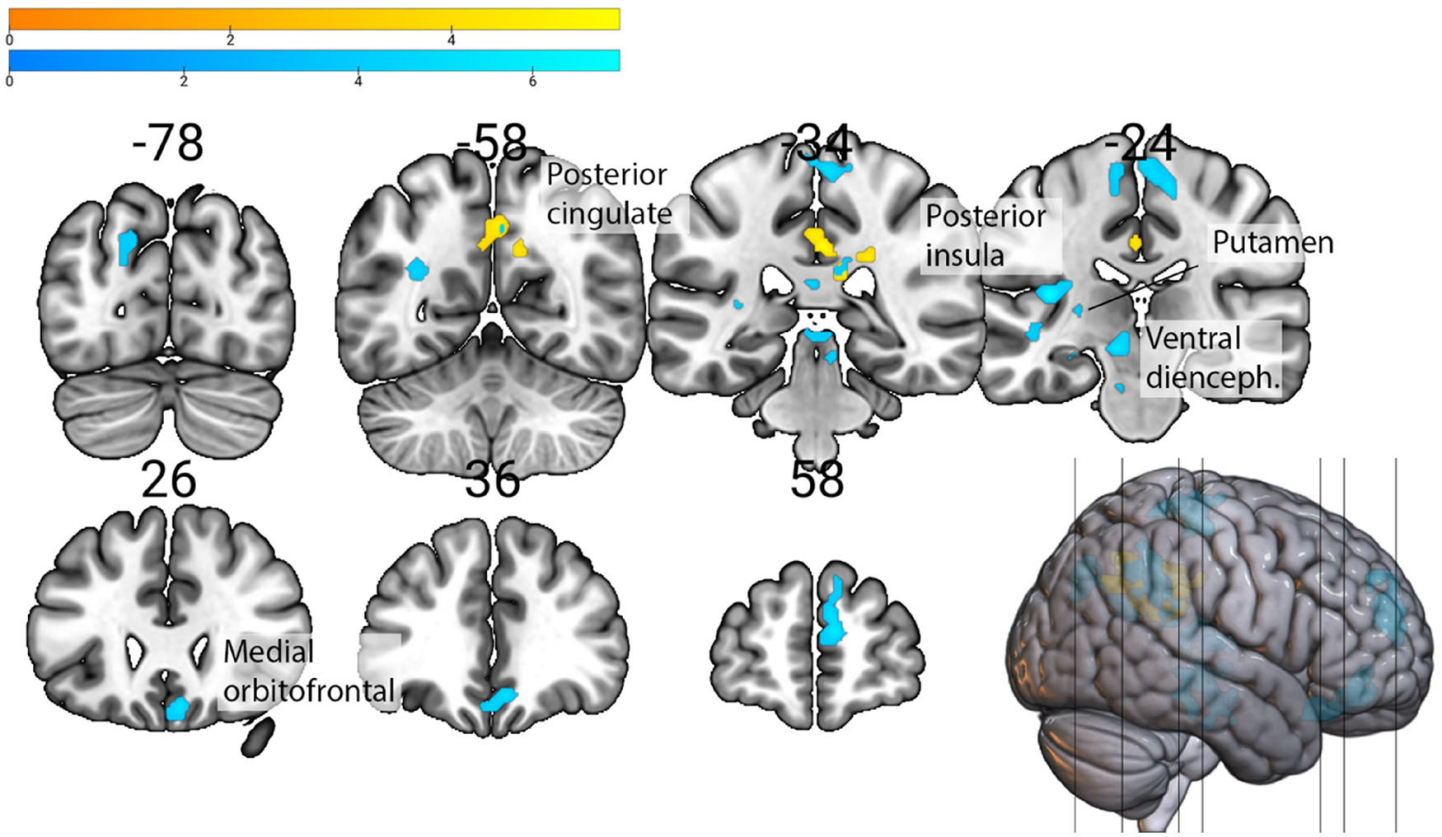
Brain areas showing higher activity in association with Prosocial Interactions during anticipation of **(a)** Monetary (yellow) and **(b)** Social (blue) rewards compared to Neutral rewards condition (*n* = 19).

**FIGURE 4:**
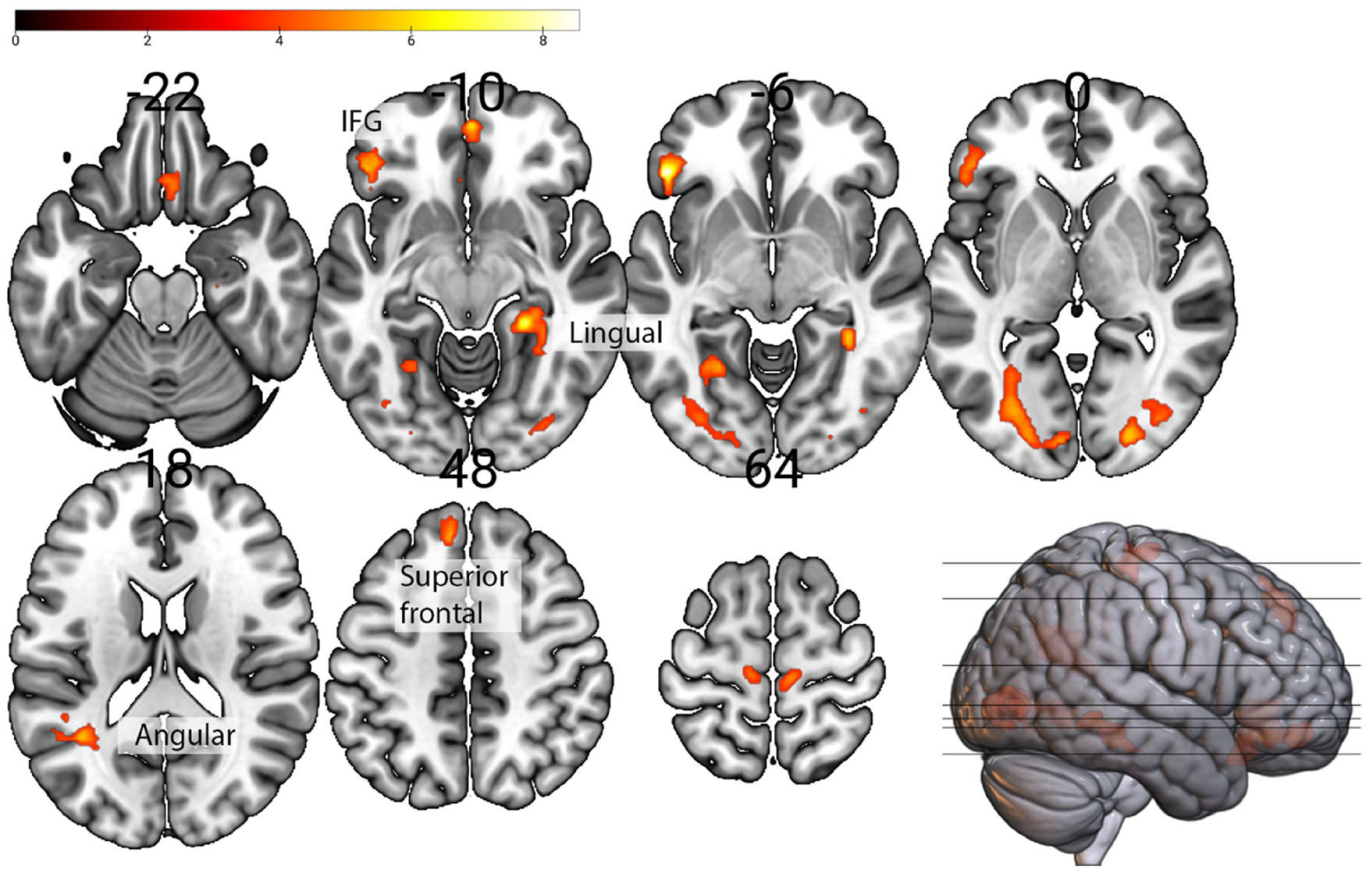
Brain areas showing higher activity in association with Prosocial Interactions during anticipation of Social rewards, compared to Monetary reward condition (axial view, z plane) (*n* = 19).

Of the four SRQ subscales of interest (Supplementary Table 3), the Prosocial Interactions scores showed the strongest positive association with anticipation of both monetary and social rewards over Neutral rewards. Specifically, it was associated with higher activity in the right posterior cingulate when anticipating monetary rewards, and with higher activity in the left posterior insula, putamen, hippocampus, cingulate, and right medial superior frontal and medial frontal gyri when anticipating social rewards, both over neutral rewards (Figure 3). Furthermore, the prosocial Interactions subscale scores were associated with higher activity in the left inferior and superior frontal areas, superior temporal areas, and right parahippocampus and medial frontal gyrus when anticipating social (over Monetary) rewards (Figure 4).

In the consumption phase (Supplementary Table 4), the Prosocial Interactions scores correlated with higher activity in the right middle superior frontal to anterior cingulate areas and with lower activity in the left superior parietal gyrus during monetary reward consumption (over baseline). The Prosocial Interactions scores were also associated with lower activity in the middle occipital areas and temporal areas, mostly left‐sided, during social reward consumption (over baseline) and in the thalamus bilaterally, right caudate nucleus, left pallidum and putamen, right temporal areas, and left IFG, parahippocampus, fusiform gyrus, and further occipital and parietal areas during the neutral reward condition.

**FIGURE 5:**
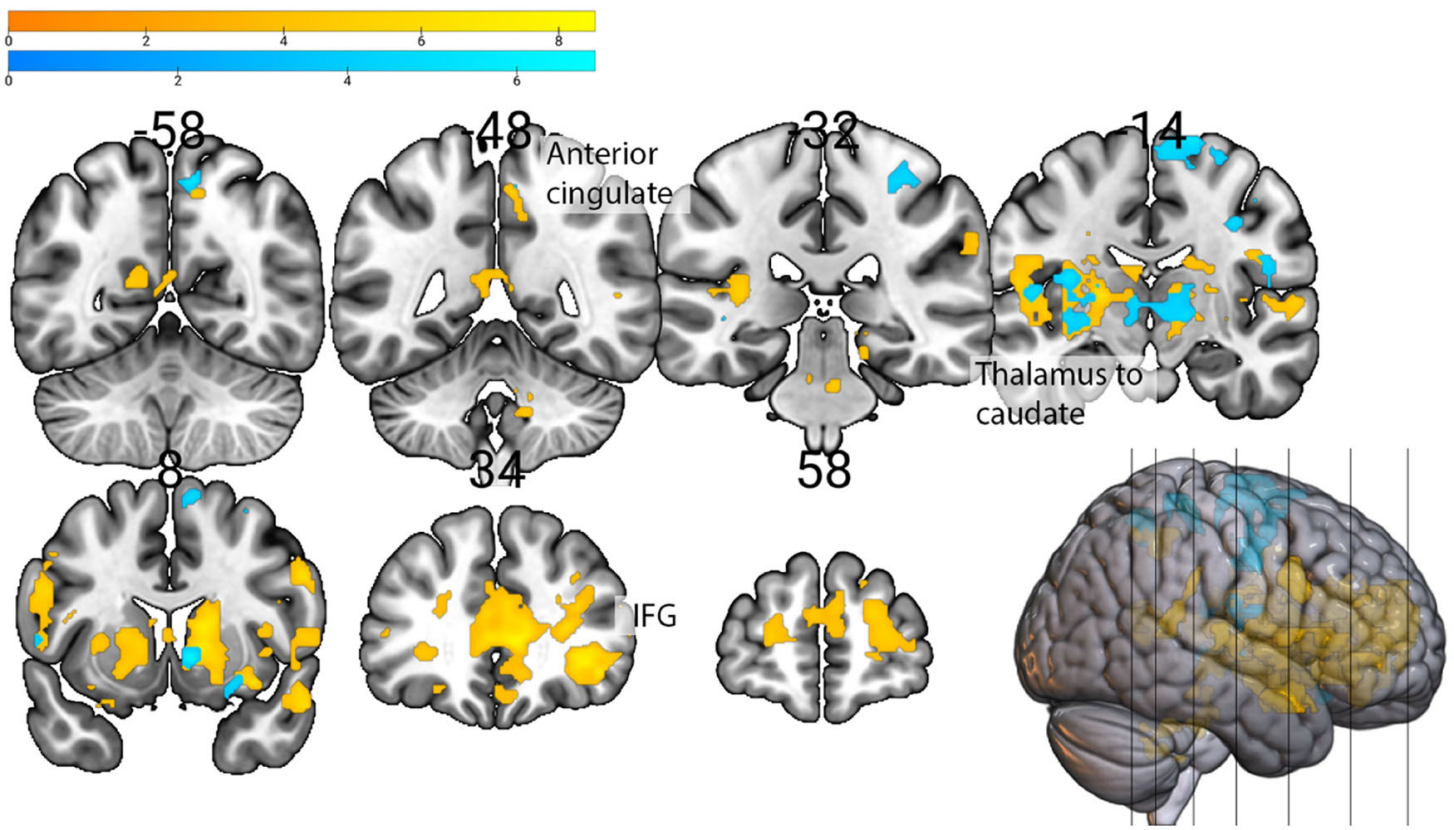
Prosocial Interactions subscale scores positively associated with higher activity in brain areas during consumption of **(a)** Monetary (yellow) and **(b)** Social rewards (blue), both compared to Neutral rewards consumption (*n* = 19).

Consumption of monetary rewards compared to neutral (Figure 5), in individuals with higher Prosocial Interactions scores, showed higher activation in the right anterior cingulate and orbital gyrus, IFG bilaterally, and left caudate nucleus and thalamus. During the consumption of social rewards compared to neutral, this showed higher activation in the right caudate, thalamus, frontal areas, and bilaterally in the operculum, insula, and putamen (Figure 5).

In more prosocial individuals, monetary compared to social reward consumption led to higher activity in the middle frontal gyrus bilaterally, the right anterior cingulate, and the left superior temporal gyrus.

## 5 Discussion

This study examined neural mechanisms involved in processing monetary and social rewards during anticipation and consumption phases. To characterize associations between neural activations and subjective social reward preferences, it examined the relationship between anticipation and consumption of different reward types and self‐reported social reward preferences.

### 5.1 Task‐related Activations: Anticipation and Consumption of Monetary and Social Rewards

We observed that the anticipation phase for all reward types activated the dorsal striatum, the salience network areas, the IFG, and supplementary motor areas, in concordance with previous findings (Oldham et al., 2018). In contrast, the consumption phase activated mainly posterior cortical areas—the fusiform and occipital gyri, and the IFG. These activations are partly consistent with existing studies of reward consumption (Oldham et al., 2018). Posterior cortical activation might suggest the involvement of supplementary areas during reward processing, perhaps indicating that these areas may respond to the reward value of visual stimuli (Künig et al., 2000). The thalamus was also activated during the consumption of Monetary and Social rewards. It is involved in the reward consumption phase by further reinforcing behavior rewarded by money compared to a simple reinforcer (i.e., word OK) (Thut et al., 1997). Differences in activity patterns for Monetary and Social reward consumption were primarily observed in the fusiform gyrus, possibly reflecting the greater visual complexity and social content of the social reward stimuli (i.e., more avatars displayed and moving).

### 5.2 Subjective Preference for Social Rewards and Neural Activations for Monetary and Social Rewards

The present study found links between (SRQ‐indexed) subjective social reward preferences and neural processing of monetary and social rewards. Specifically, higher preference for prosocial interactions, which reflects the preference to draw rewards from reciprocal relationships (Foulkes et al., 2014), was associated with stronger activity in the right posterior cingulate when anticipating a monetary reward. This may indicate that, for individuals who value prosocial behavior, the prospect of monetary gain may engage brain regions associated with self‐referential thought and social cognition, as the posterior cingulate is implicated in these processes (Margulies et al., 2009).

When anticipating Social rewards compared to monetary rewards (and neutral stimuli), preference for prosocial interactions was associated with stronger activity in the striatum and the salience network in the left hemisphere. The striatum is a key region involved in reward processing and motivation (Delgado, 2007), indicating that social rewards may be particularly motivating and rewarding for individuals who report subjective feelings of reward from prosocial interactions. The engagement of the salience network, which includes the anterior insula and dorsal anterior cingulate cortex, suggests that social rewards are given a high priority and are processed as significant stimuli for individuals who report greater subjective preference for prosocial rewards (Seeley et al., 2007). While monetary rewards activated the striatum in relation to prosociality, its involvement and the salience network highlight a unique aspect of social reward preference for those with prosocial tendencies (Izuma et al., 2008). These results add to existing literature by demonstrating hemispheric differences in the reward system, suggesting that social and monetary rewards may be integrated with personal values in complex and individual‐specific ways (Foulkes et al., 2014).

Different activation patterns related to prosociality were found during the consumption phase of various types of rewards. Highly prosocial individuals activated mainly the frontal and superior temporal regions, dorsal striatum and thalamus when consuming monetary rewards as opposed to Neutral stimuli. This pattern of activation is consistent with the involvement of these brain regions in processing monetary rewards (Delgado et al., 2000; Knutson, Adams, et al., 2001). The dorsal striatum (caudate nucleus and putamen) and thalamus are part of the brain's reward circuit and are involved in the processing and anticipation of rewards (Schultz, 2015). These findings suggest that highly prosocial individuals might also draw strong rewarding experiences from monetary incentives. On the other hand, the task's monetary reward featured a “human avatar holding a jar with coins,” which could indicate a certain social component of the Monetary reward implicated in this experiment.

The consumption of social rewards over neutral stimuli in those with higher prosociality was strongly associated with higher activity in the putamen. The putamen is part of the dorsal striatum and is involved in social reward processing, such as positive social interactions and acceptance (Izuma et al., 2008). This finding suggests that highly prosocial individuals derive significant reward value from social rewards compared to neutral rewards, replicating previous research that has shown the involvement of the putamen in the processing of social rewards, adding that this may be associated with subjective prosocial reward experiences.

We observed no associations between Negative Social Potency, Passivity, Sexual Reward, or Sociability domains of the SRQ and brain activity during the anticipation or consumption phase of social/monetary reward types. This was most likely explained by using a limited number of reward scenarios in the MSDIT task that would have been specifically relevant to these domains. Moreover, the aforementioned SRQ domains showed limited score ranges or low scores in this sample.

### 5.3 Potential Clinical Research Implications

The present findings demonstrate potential links between subjective experiences of interpersonal pleasure and neural responses to social rewards. From a clinical perspective, the subjective experience of atypical social reward (e.g., gratification from causing conflict, seeking social withdrawal, etc.) resulting in changes in enjoyment of interpersonal connections is a potential transdiagnostic feature of psychopathology (Barkus, 2021; Barkus and Badcock, 2019; Gooding and Pflum, 2022; Marder and Galderisi, 2017). Adjustments in the subjective experience of social reward contribute to psychosocial distress, social anhedonia, social motivation, and atypical interpersonal behavior within patient groups (Abel et al., 2023; Fulford et al., 2018; Llerena et al., 2012; Michelini et al., 2021). Alongside this, there is increasing evidence that neuropsychological processes involved in social reward preference might be interrupted or adjusted in those with mental health difficulties, as seen in studies of reward anticipation and consumption in psychosis (Chan et al., 2024; Mow et al., 2020; Shackman et al., 2025), depression (Daniels et al., 2025; Höflich et al., 2019; Zhang et al., 2022), and anxiety disorders (Aldridge‐Waddon et al., 2020; Cremers et al., 2015; Richey et al., 2014). The results of the present study highlight the importance of future clinical studies considering associations between subjective experience of reward and neuropsychological correlates. This may help to infer whether the associations seen here translate to clinical groups and, if so, whether subjective preferences for different types of social reward (or perhaps less of a preference towards social rewards) in patient groups are linked to similar neural activation patterns as those observed here.

### 5.4 Limitations

The present study had limitations in its design and implementation, which future research should address. Participants' mental health status was based on self‐report only, which is a common practice in this type of research. Unvalidated avatar reward stimuli were used as monetary and social rewards (i.e., no actual reward was received), potentially limiting the applicability and generalizability of the presented findings (Aldridge‐Waddon et al., 2022). While the SRQ is a well‐established measure, it may be oversimplifying complex interpersonal processes (Foulkes et al., 2014) and the multifactorial nature of social reward preferences (Matyjek et al., 2020). Future studies could corroborate current findings by including additional measures of (social) reward preferences—e.g., the Sensitivity to Punishment and Sensitivity to Reward Questionnaire (Torrubia et al., 2001) to verify associations between social reward preferences and neural responses during the MSIDT. Furthermore, stimuli denoting different types of social reward (e.g., admiration, sociability) were included but grouped as “social rewards.” As shown with SRQ responses, various types of social reward can be experienced differently, and future research should examine neural mechanisms involved in these processing differences. The reliability of the findings is also limited by the small participant sample size and the relatively reduced power of some statistical comparisons.

## 6 Conclusions

The present findings underline the importance of considering individual differences in social reward preferences, especially in relation to prosocial tendencies. A better understanding of associations between subjective individual differences in reward experiences, for example, trait prosociality, and neural responses during reward processing might inform more bespoke models of social reward processing. This could also contribute to better‐targeted interventions to increase prosocial behaviors in clinical groups where social reward mechanisms are thought to be altered (Aldridge‐Waddon et al., 2020). Identifying links between subjective social reward experiences and neural mechanisms of reward processing can enhance existing conceptualizations of reward‐specific anticipation and consumption.

## Author Contributions

**Martina Vanova**: conceptualization, data curation, formal analysis, investigation, methodology, project administration, visualization, writing – original draft, writing – review and editing. **Luke Aldridge‐Waddon**: conceptualization, methodology, project administration, writing – original draft, writing – review and editing. **Ignazio Puzzo**: conceptualization, methodology, supervision, writing – review and editing. **Veena Kumari**: conceptualization, formal analysis, funding acquisition, methodology, resources, supervision, writing – review and editing.

## Ethics Statement

This research was approved by the College of Health, Medicine and Life Sciences Research Ethics Committee, Brunel University London (Reference: 16789‐MHR‐May/2019–19042‐2). All participants gave written informed consent after the study procedures had been explained to them.

## Conflicts of Interest

The authors declare no conflicts of interest.

## Peer Review

The peer review history for this article is available at https://publons.com/publon/10.1002/brb3.70776

## Supporting information




**Supplementary Tables**: brb370776‐sup‐0001‐Tables.docx

## Data Availability

All stimuli, behavioral data, and the fMRI data with analysis codes will be available from the Brunel University London research repository (https://doi.org/10.17633/rd.brunel.29852201). Legal copyright restrictions prevent public archiving of the SRQ, which can be obtained from the copyright holders in the cited references. We reported how we determined our sample size, all data exclusions, all inclusion/exclusion criteria established prior to data analysis, all manipulations, and all measures in this study. No part of this study (procedures or analyses) was pre‐registered prior to the research being conducted.
