# Supplementary material for: Neural Correlates of Reward Processing: Impact of Individual Differences in Preference for Prosocial Interactions
Source: Brain Behav. 2025 Sep 8;15(9):e70776. doi: 10.1002/brb3.70776 (PMC12417630; doi:10.1002/brb3.70776)
Supplement: Supplementary file 1 — Supplementary Tables: brb370776‐sup‐0001‐Tables.docx [file BRB3-15-e70776-s001.docx]

Supplementary Table 1. Task-related activations (MSIDT) (T-contrasts) during the reward anticipation phase across the entire sample (*n* = 20) [height threshold family-wise error (FWE) corrected *p* < .05; extent threshold = 2 voxels].

|  |  |  | **cluster** | | **peak** | | **MNI (mm)** | | |
| --- | --- | --- | --- | --- | --- | --- | --- | --- | --- |
| **Area** | **BA** | **side** | **p (FWE)** | **K (equiv.)** | **p (FWE)** | **T** | **x** | **y** | **z** |
|  |  |  |  |  |  |  |  |  |  |
| ***Monetary > rest*** | | | | | | | | | |
| Putamen (Pallidum) |  | L | < .000 | 187 | 0.002 | 9.05 | -20 | 4 | -4 |
|  |  |  |  |  |  |  |  |  |  |
| Anterior insula | 47 | R | < .000 | 155 | 0.046 | 6.96 | 46 | 20 | -2 |
| Inferior frontal (opercularis) to ant insula |  |  |  |  | 0.349 | 5.61 | 48 | 10 | 2 |
| Inferior frontal (opercularis) |  |  |  |  | 0.593 | 5.16 | 56 | 18 | 4 |
|  |  |  |  |  |  |  |  |  |  |
| Precentral | 6 | L | 0.004 | 94 | 0.067 | 6.72 | -36 | -12 | 52 |
|  |  |  |  |  |  |  |  |  |  |
| Supplementary motor area | 6 | R | < .000 | 390 | 0.203 | 5.99 | 4 | 8 | 58 |
|  |  |  |  |  | 0.219 | 5.94 | 10 | 12 | 50 |
|  |  | L |  |  | 0.381 | 5.54 | -2 | 4 | 52 |
|  |  |  |  |  |  |  |  |  |  |
| Pallidum to Putamen |  | R | 0.001 | 118 | 0.309 | 5.7 | 18 | 8 | 2 |
|  |  |  |  |  |  |  |  |  |  |
| ***Social > rest*** | | | | | | | | | |
| Supplementary motor area | 6 | R | < .000 | 366 | 0.019 | 7.58 | 6 | 12 | 54 |
|  |  |  |  |  | 0.044 | 7.02 | 0 | 2 | 58 |
|  |  |  |  |  |  |  |  |  |  |
| Inferior frontal (opercularis) | 13 | R | 0.014 | 63 | 0.106 | 6.45 | 50 | 18 | -2 |
| Insula |  |  |  |  | 0.519 | 5.31 | 42 | 12 | 2 |
|  |  |  |  |  |  |  |  |  |  |
| Putamen |  | R | 0.030 | 50 | 0.391 | 5.54 | 18 | 10 | -2 |
|  |  |  |  |  |  |  |  |  |  |
| ***Neutral > rest*** | | | | | | | | | |
| Supplementary motor area | 6 | L | < .000 | 475 | 0.009 | 8.19 | -4 | 14 | 48 |
|  |  | R |  |  | 0.028 | 7.46 | 4 | 10 | 56 |
|  |  |  |  |  | 0.037 | 7.27 | 6 | 14 | 48 |
|  |  |  |  |  |  |  |  |  |  |
| Inferior frontal (opercularis) | 44 | R | 0.030 | 43 | 0.041 | 7.21 | 56 | 14 | 14 |

*Note*: Anticipation and consumption (won) phase contrasts: Monetary > rest; Social >rest; Neutral > rest; Monetary > neutral; Social > neutral; Monetary Social; Social > Monetary.

Supplementary Table 2. Task-related activations (MSIDT) (T-contrasts) during the reward consumption phase across the entire sample (*n* = 20) [height threshold family-wise error (FWE) corrected *p* < .05; extent threshold = 2 voxels].

|  |  |  | **cluster** | | **peak** | | **MNI (mm)** | | |
| --- | --- | --- | --- | --- | --- | --- | --- | --- | --- |
| **Area** | **BA** | **side** | **p (FWE)** | **K (equiv.)** | **p (FWE)** | **T** | **x** | **y** | **z** |
|  |  |  |  |  |  |  |  |  |  |
| ***Monetary won > rest*** | | | | | | | | | |
| Fusiform | 37/19 | R | < .000 | 2932 | < .000 | 14.41 | 32 | -50 | -12 |
| Inferior temporal |  |  |  |  | < .000 | 13.58 | 42 | -56 | -12 |
| Middle temporal |  |  |  |  | < .000 | 13.2 | 44 | -64 | 2 |
|  |  |  |  |  |  |  |  |  |  |
| Middle occipital | 37/19 | L | < .000 | 2799 | < .000 | 14.16 | -44 | -68 | 4 |
| Fusiform |  |  |  |  | < .000 | 10.42 | -32 | -54 | -14 |
|  |  |  |  |  | < .000 | 9.96 | -46 | -58 | -16 |
|  |  |  |  |  |  |  |  |  |  |
| Inferior frontal (triangularis) | 9 | R | < .000 | 311 | 0.014 | 7.87 | 48 | 22 | 28 |
| Inferior frontal (opercularis) |  |  |  |  | 0.117 | 6.49 | 48 | 12 | 30 |
| Precentral |  |  |  |  | 0.172 | 6.23 | 48 | 12 | 40 |
|  |  |  |  |  |  |  |  |  |  |
| Middle occipital | 14 | R | < .000 | 179 | 0.015 | 7.82 | 30 | -66 | 36 |
|  |  |  |  |  | 0.677 | 5.15 | 30 | -60 | 46 |
|  |  |  |  |  |  |  |  |  |  |
| Thalamus |  | R | 0.035 | 42 | 0.298 | 5.85 | 22 | -26 | -2 |
|  |  |  |  |  |  |  |  |  |  |
| ***Social won > rest*** | | | | | | | | | |
| Middle temporal | 37/19 | R | < .000 | 3978 | < .000 | 13.29 | 48 | -68 | 0 |
| Fusiform |  |  |  |  | < .000 | 10.89 | 42 | -44 | -18 |
|  |  |  |  |  | < .000 | 10.78 | 28 | -72 | -12 |
|  |  |  |  |  |  |  |  |  |  |
| Middle occipital | 37/19 | L | < .000 | 2258 | < .000 | 11.25 | -46 | -68 | 6 |
|  |  |  |  |  | < .000 | 11.21 | -30 | -86 | 4 |
|  |  |  |  |  | 0.003 | 8.82 | -36 | -82 | 0 |
|  |  |  |  |  |  |  |  |  |  |
| Middle frontal | 9 | R | < .000 | 576 | 0.001 | 9.44 | 38 | 6 | 40 |
| Inferior frontal (triangularis) |  |  |  |  | 0.032 | 7.28 | 46 | 24 | 26 |
|  |  |  |  |  | 0.215 | 6.03 | 54 | 36 | 2 |
|  |  |  |  |  |  |  |  |  |  |
| Thalamus |  | L | 0.049 | 39 | 0.077 | 6.72 | -20 | -26 | -2 |
|  |  |  |  |  |  |  |  |  |  |
| Inferior frontal (triangularis) |  | L | 0.046 | 40 | 0.589 | 5.25 | -52 | 32 | 14 |
|  |  |  |  |  | 0.653 | 5.14 | -48 | 30 | 4 |
|  |  |  |  |  |  |  |  |  |  |
| ***Neutral won > rest*** | | | | | | | | | |
| Fusiform | 37/19 | R | < .000 | 2434 | < .000 | 12.59 | 34 | -52 | -12 |
|  |  |  |  |  | < .000 | 9.9 | 38 | -44 | -16 |
| Middle temporal |  |  |  |  | 0.001 | 9.53 | 50 | -68 | 0 |
|  |  |  |  |  |  |  |  |  |  |
| Middle occipital | 37/19 | L | < .000 | 1238 | < .000 | 9.98 | -42 | -76 | 6 |
|  |  |  |  |  | 0.002 | 9.19 | -30 | -86 | 4 |
|  |  |  |  |  | 0.002 | 9.03 | -36 | -82 | -2 |
|  |  |  |  |  |  |  |  |  |  |
| Lingual | 17 | L | < .000 | 145 | 0.049 | 7.01 | -6 | -80 | 4 |
| Calcarine |  |  |  |  | 0.593 | 5.24 | -14 | -76 | 10 |
|  |  |  |  |  |  |  |  |  |  |
| Thalamus | 30/27 | L | 0.021 | 53 | 0.157 | 6.24 | -22 | -24 | -2 |
| Parahippocampal |  |  |  |  | 0.403 | 5.57 | -18 | -38 | -6 |
| Lingual |  |  |  |  | 0.851 | 4.79 | -20 | -46 | -8 |
|  |  |  |  |  |  |  |  |  |  |
| Calcarine | 17 | R | 0.009 | 68 | 0.459 | 5.47 | 6 | -86 | 4 |
|  |  |  |  |  | 0.530 | 5.34 | 10 | -82 | 10 |
|  |  |  |  |  |  |  |  |  |  |
| ***Monetary won > neutral won*** | | | | | | | | | |
| Inferior temporal | 37/39 | L | < .000 | 453 | 0.006 | 8.32 | -44 | -62 | -6 |
| Middle temporal |  |  |  |  | 0.006 | 8.31 | -54 | -50 | 6 |
| Inferior occipital |  |  |  |  | 0.030 | 7.24 | -48 | -62 | -16 |
|  |  |  |  |  |  |  |  |  |  |
| Inferior occipital | 18 | L | 0.003 | 98 | 0.013 | 7.8 | -28 | -86 | -2 |
| Fusiform |  |  |  |  | 0.362 | 5.58 | -30 | -80 | -16 |
|  |  |  |  |  |  |  |  |  |  |
| Inferior occipital | 18 | R | 0.014 | 65 | 0.112 | 6.39 | 32 | -86 | 0 |
|  |  |  |  |  |  |  |  |  |  |
| ***Social won > neutral won*** | | | | | | | | | |
| Middle temporal | 22/13 | R | < .000 | 1404 | < .000 | 11.67 | 48 | -64 | 0 |
|  |  |  |  |  | 0.001 | 9.69 | 46 | -58 | 6 |
| Superior temporal |  |  |  |  | 0.004 | 8.71 | 64 | -36 | 16 |
|  |  |  |  |  |  |  |  |  |  |
| Middle occipital | 19/18 | L | < .000 | 408 | 0.001 | 9.3 | -18 | -90 | -2 |
| Lingual |  |  |  |  | 0.002 | 9.13 | -22 | -84 | -12 |
| Middle occipital |  |  |  |  | 0.004 | 8.73 | -26 | -86 | 8 |
|  |  |  |  |  |  |  |  |  |  |
| Middle occipital | 39/37 | L | < .000 | 1306 | 0.002 | 9.02 | -44 | -66 | 4 |
| Middle temporal |  |  |  |  | 0.011 | 8.13 | -54 | -56 | 4 |
| Fusiform |  |  |  |  | 0.014 | 7.95 | -42 | -52 | -10 |
|  |  |  |  |  |  |  |  |  |  |
| Lingual | 18/17 | R | < .000 | 365 | 0.027 | 7.53 | 22 | -88 | -6 |
| Fusiform |  |  |  |  | 0.131 | 6.48 | 28 | -78 | -4 |
|  |  |  |  |  | 0.282 | 5.96 | 32 | -70 | -8 |
|  |  |  |  |  |  |  |  |  |  |
| Fusiform | 20 | R | 0.004 | 72 | 0.056 | 7.04 | 40 | -38 | -20 |
|  |  |  |  |  |  |  |  |  |  |
| Middle temporal | 21 | R | 0.009 | 58 | 0.164 | 6.33 | 52 | -8 | -16 |
| Superior temporal |  |  |  |  | 0.845 | 4.91 | 52 | -12 | -8 |
|  |  |  |  |  |  |  |  |  |  |
| ***Monetary won > social won*** | | | | | | | | | |
| Fusiform | 37 | L | 0.024 | 50 | 0.010 | 8.09 | -28 | -40 | -18 |
|  |  |  |  |  |  |  |  |  |  |
| **Social won > monetary won** | | | | | | | | | |
| Calcarine | 18/17 | R | < .000 | 600 | < .000 | 11.96 | 14 | -88 | 4 |
| Lingual |  |  |  |  | 0.007 | 8.32 | 14 | -80 | -8 |
| Superior occipital |  |  |  |  | 0.073 | 6.76 | 20 | -86 | 18 |
|  |  |  |  |  |  |  |  |  |  |
| Superior occipital | 18/17 | L | < .000 | 547 | 0.004 | 8.73 | -14 | -92 | 4 |
| Middle occipital |  |  |  |  | 0.030 | 7.35 | -18 | -88 | 16 |
| Cuneus |  |  |  |  | 0.115 | 6.47 | -10 | -80 | 16 |
|  |  |  |  |  |  |  |  |  |  |
| Middle temporal | 22/39 | R | < .000 | 455 | 0.038 | 7.18 | 50 | -66 | 2 |
| Superior temporal |  |  |  |  | 0.062 | 6.86 | 60 | -36 | 16 |
| Middle temporal |  |  |  |  | 0.070 | 6.78 | 52 | -50 | 10 |
|  |  |  |  |  |  |  |  |  |  |
| Fusiform | 37 | R | 0.024 | 50 | 0.170 | 6.2 | 40 | -42 | -20 |
|  |  |  |  |  |  |  |  |  |  |
| Fusiform | 19 | R | 0.048 | 39 | 0.191 | 6.13 | 26 | -70 | -4 |
|  |  |  |  |  | 0.603 | 5.23 | 32 | -74 | -10 |

Supplementary Table 3. Relationships (negative [-] and positive [+] associations) between task-related activations (MSIDT) and reward processing traits (SRQ) during the reward anticipation phase (height threshold *p* < .001 uncorrected), (*n* = 19).

| **Contrast** |  |  | **cluster** | | | **peak** | | **MNI (mm)** | | |
| --- | --- | --- | --- | --- | --- | --- | --- | --- | --- | --- |
| **Area** | **BA** | **side** | **p (FWE)** | **K (equiv.)** | **p (unc.)** | **T** | **p (unc.)** | **x** | **y** | **z** |
|  |  |  |  |  |  |  |  |  |  |  |
| ***Monetary > Rest (Sexual+)*** | | | | | | | | | | |
| Precuneus | 31 | R | 0.045 | 153 | 0.004 | 5.66 | < .001 | 16 | -56 | 22 |
|  |  |  |  |  |  |  |  |  |  |  |
| ***Neutral > Rest (Prosocial-)*** | | | | | | | | | | |
| Inferior parietal | 31 | R | 0.003 | 190 | < .001 | 6.03 | < .001 | 26 | -44 | 30 |
|  |  |  |  |  |  | 5.2 | < .001 | 36 | -44 | 32 |
|  |  |  |  |  |  | 4.78 | < .001 | 24 | -42 | 44 |
|  |  |  |  |  |  |  |  |  |  |  |
| Superior intraparietal | 40 | L | < .001 | 300 | < .001 | 5.62 | < .001 | -24 | -42 | 42 |
|  |  |  |  |  |  | 5.16 | < .001 | -28 | -50 | 36 |
|  |  |  |  |  |  | 4.48 | < .001 | -34 | -50 | 50 |
|  |  |  |  |  |  |  |  |  |  |  |
| ***Monetary > Neutral (Prosocial+)*** | | | | | | | | | | |
| Posterior cingulate | 31 | R | < .001 | 668 | < .001 | 5.63 | < .001 | 2 | -34 | 34 |
| Precuneus |  | L |  |  |  | 5.35 | < .001 | -4 | -52 | 32 |
|  |  | R |  |  |  | 5.32 | < .001 | 22 | -34 | 28 |
|  |  |  |  |  |  |  |  |  |  |  |
| ***Social > Neutral (Prosocial+)*** | | | | | | | | | | |
| Posterior insula | 13 | L | 0.014 | 308 | 0.002 | 7.14 | < .001 | -38 | -24 | 14 |
| Putamen |  |  |  |  |  | 5.5 | < .001 | -24 | -18 | 8 |
|  |  |  |  |  |  | 5.2 | < .001 | -32 | -32 | 10 |
|  |  |  |  |  |  |  |  |  |  |  |
| Paracentral lobule to supplementary motor | 6 | R | 0.005 | 391 | 0.001 | 6.86 | < .001 | 6 | -26 | 62 |
|  |  | L |  |  |  | 5.31 | < .001 | -10 | -22 | 66 |
|  |  | R |  |  |  | 5 | < .001 | 6 | -38 | 66 |
|  |  |  |  |  |  |  |  |  |  |  |
| Medial superior frontal | 9/10 | R | 0.013 | 316 | 0.002 | 6.01 | < .001 | 8 | 58 | 14 |
|  |  |  |  |  |  | 5.52 | < .001 | 16 | 50 | 36 |
|  |  |  |  |  |  | 5.04 | < .001 | 4 | 54 | 36 |
|  |  |  |  |  |  |  |  |  |  |  |
| Ventral diencephalon to hippocampus |  | L | 0.007 | 363 | 0.001 | 5.89 | < .001 | -10 | -24 | -10 |
| Internal capsule |  |  |  |  |  | 5.58 | < .001 | -14 | -16 | -16 |
|  |  |  |  |  |  | 5.44 | < .001 | -10 | -10 | -26 |
|  |  |  |  |  |  |  |  |  |  |  |
| Cingulate | 31 | L | < .001 | 726 | < .001 | 5.76 | < .001 | -2 | -36 | 16 |
| Precuneus |  | R |  |  |  | 5.15 | < .001 | 16 | -44 | 38 |
|  |  | L |  |  |  | 4.87 | < .001 | -6 | -46 | 36 |
|  |  |  |  |  |  |  |  |  |  |  |
| Rectus | 11 | R | 0.025 | 268 | 0.003 | 5.63 | < .001 | 6 | 26 | -18 |
| Medial orbitofrontal |  |  |  |  |  | 5.44 | < .001 | 6 | 36 | -14 |
|  |  | L |  |  |  | 5.02 | < .001 | -2 | 34 | -20 |
|  |  |  |  |  |  |  |  |  |  |  |
| Parietooccipital | 7 | L | 0.008 | 347 | 0.001 | 4.79 | < .001 | -16 | -74 | 40 |
|  |  |  |  |  |  | 4.52 | < .001 | -18 | -76 | 32 |
| Angular gyrus |  |  |  |  |  | 4.42 | < .001 | -30 | -58 | 22 |
|  |  |  |  |  |  |  |  |  |  |  |
| ***Social > Monetary (Prosocial +)*** | | | | | | | | | | |
| Inferior frontal (triangularis) | 47 | L | 0.003 | 237 | < .001 | 8.98 | < .001 | -48 | 32 | -6 |
| Superior orbital |  |  |  |  |  | 4.6 | < .001 | -40 | 36 | -16 |
|  |  |  |  |  |  |  |  |  |  |  |
| Parahippocampal | 36/19 | R | 0.003 | 229 | < .001 | 7.72 | < .001 | 26 | -36 | -10 |
|  |  |  |  |  |  | 6.03 | < .001 | 34 | -44 | -8 |
|  |  |  |  |  |  | 4.01 | 0.001 | 24 | -20 | -22 |
|  |  |  |  |  |  |  |  |  |  |  |
| Medial frontal (gyrus rectus) | 11 | R | < .001 | 389 | < .001 | 6.75 | < .001 | 0 | 50 | -12 |
| Medial frontal orbital |  |  |  |  |  | 6.42 | < .001 | 8 | 36 | -14 |
| Subcallosal |  |  |  |  |  | 6.24 | < .001 | 4 | 26 | -20 |
|  |  |  |  |  |  |  |  |  |  |  |
| Lateral occipital | 13 | R | 0.004 | 221 | < .001 | 5.81 | < .001 | 24 | -86 | 0 |
|  |  |  |  |  |  | 4.89 | < .001 | 36 | -80 | 0 |
|  |  |  |  |  |  | 4.89 | < .001 | 28 | -88 | -8 |
|  |  |  |  |  |  |  |  |  |  |  |
| Superior temporal | 39 | L | < .001 | 584 | < .001 | 5.77 | < .001 | -38 | -54 | 18 |
|  |  |  |  |  |  | 5.67 | < .001 | -48 | -52 | 30 |
| Angular |  |  |  |  |  | 5.39 | < .001 | -42 | -64 | 28 |
|  |  |  |  |  |  |  |  |  |  |  |
| Superior frontal | 8 | L | 0.018 | 162 | 0.001 | 5.57 | < .001 | -8 | 38 | 48 |
|  |  |  |  |  |  | 5.46 | < .001 | -8 | 44 | 40 |
|  |  |  |  |  |  | 4.47 | < .001 | -6 | 34 | 56 |
|  |  |  |  |  |  |  |  |  |  |  |
| Calcarine | 17/19 | L | < .001 | 538 | < .001 | 5.53 | < .001 | -10 | -92 | -2 |
|  |  |  |  |  |  | 5.43 | < .001 | -28 | -76 | 0 |
| Lingual |  |  |  |  |  | 5.43 | < .001 | -24 | -56 | -6 |
|  |  |  |  |  |  |  |  |  |  |  |
| Medial precentral | 6 | R | 0.002 | 252 | < .001 | 4.93 | < .001 | 8 | -28 | 64 |
| Paracentral |  | L |  |  |  | 4.72 | < .001 | -14 | -18 | 68 |
|  |  | R |  |  |  | 4.67 | < .001 | 12 | -16 | 68 |

Supplementary Table 4. Relationships (negative [-] and positive [+] associations) between task-related activations (MSIDT) and reward processing traits (SRQ) during the reward consumption phase (height threshold *p* < .001 uncorrected), (*n* = 19).

|  |  |  | **cluster** | | | **peak** | | **MNI (mm)** | | |
| --- | --- | --- | --- | --- | --- | --- | --- | --- | --- | --- |
| **Area** | **BA** | **side** | **p (FWE)** | **K (equiv.)** | **p (unc.)** | **T** | **p (unc.)** | **x** | **y** | **z** |
|  |  |  |  |  |  |  |  |  |  |  |
| ***Monetary won > Rest (Prosocial+)*** | | | | | | | | | | |
| Medial superior frontal to cingulate | 9 | R | 0.015 | 158 | 0.001 | 5.54 | < .001 | 4 | 48 | 18 |
|  |  |  |  |  |  | 4.3 | < .001 | 18 | 54 | 28 |
|  |  |  |  |  |  |  |  |  |  |  |
| Superior frontal | 10 | R | 0.039 | 126 | 0.003 | 5.02 | < .001 | 22 | 54 | 16 |
|  |  |  |  |  |  | 4.35 | < .001 | 22 | 62 | 18 |
| Mid frontal |  |  |  |  |  | 4.34 | < .001 | 26 | 42 | 20 |
|  |  |  |  |  |  |  |  |  |  |  |
| ***Monetary won > Rest (Prosocial-)*** | | | | | | | | | | |
| Superior parietal to precuneus | 7 | L | 0.035 | 130 | 0.002 | 5.88 | < .001 | -24 | -50 | 38 |
|  |  |  |  |  |  | 5.14 | < .001 | -22 | -56 | 44 |
|  |  |  |  |  |  |  |  |  |  |  |
| ***Social won > Rest (Prosocial-)*** | | | | | | | | | | |
| Middle occipital | 17 | L | 0.002 | 257 | < .001 | 6.98 | < .001 | -26 | -80 | 2 |
| to lingual |  |  |  |  |  | 5.54 | < .001 | -22 | -80 | 10 |
|  |  |  |  |  |  | 5.1 | < .001 | -18 | -84 | -2 |
|  |  |  |  |  |  |  |  |  |  |  |
| Superior temporal | 41 | L | < .001 | 409 | < .001 | 6.63 | < .001 | -54 | -32 | 8 |
|  |  |  |  |  |  | 5.74 | < .001 | -58 | -40 | 14 |
|  |  |  |  |  |  | 4.73 | < .001 | -52 | -46 | 14 |
|  |  |  |  |  |  |  |  |  |  |  |
| Middle temporal | 21 | R | 0.047 | 131 | 0.003 | 5.81 | < .001 | 68 | -30 | -4 |
| Superior temporal |  |  |  |  |  | 5.12 | < .001 | 68 | -32 | 8 |
|  |  |  |  |  |  |  |  |  |  |  |
| Middle occipital to parietal | 7 | L | 0.002 | 254 | < .001 | 5.32 | < .001 | -22 | -52 | 34 |
|  |  |  |  |  |  | 4.68 | < .001 | -26 | -40 | 40 |
|  |  |  |  |  |  |  |  |  |  |  |
| Inferior temporal |  | L | 0.019 | 165 | 0.001 | 5.12 | < .001 | -38 | -48 | -4 |
|  |  |  |  |  |  | 4.43 | < .001 | -44 | -56 | -12 |
|  |  |  |  |  |  |  |  |  |  |  |
| ***Neutral won > Rest (Prosocial-)*** | | | | | | | | | | |
| Thalamus to caudate |  | R | < .001 | 3017 | < .001 | 9.97 | < .001 | 10 | -18 | 0 |
| Inferior frontal (orbitalis) |  |  |  |  |  | 9.27 | < .001 | 32 | 30 | 0 |
| Thalamus to pallidum and putamen |  | L |  |  |  | 8.48 | < .001 | -12 | -20 | -2 |
|  |  |  |  |  |  |  |  |  |  |  |
| Superior temporal | 22/21 | L | < .001 | 946 | < .001 | 7.15 | < .001 | -50 | 8 | -16 |
| to middle temporal |  |  |  |  |  | 7.07 | < .001 | -38 | -48 | 16 |
|  |  |  |  |  |  | 6.69 | < .001 | -50 | 0 | -18 |
|  |  |  |  |  |  |  |  |  |  |  |
| Superior temporal | 22/21 | R | < .001 | 447 | < .001 | 6.27 | < .001 | 60 | -32 | 2 |
| to middle temporal |  |  |  |  |  | 5.93 | < .001 | 68 | -24 | -4 |
|  |  |  |  |  |  | 5.3 | < .001 | 50 | -30 | 2 |
|  |  |  |  |  |  |  |  |  |  |  |
| Inferior temporal |  | R | 0.006 | 170 | < .001 | 6.18 | < .001 | 44 | -58 | -6 |
|  |  |  |  |  |  | 5.56 | < .001 | 40 | -42 | 2 |
|  |  |  |  |  |  | 5.33 | < .001 | 40 | -52 | 0 |
|  |  |  |  |  |  |  |  |  |  |  |
| Inferior frontal (triangularis to orbitalis) | 47 | L | 0.017 | 139 | 0.001 | 6.17 | < .001 | -48 | 22 | 2 |
|  |  |  |  |  |  | 5.91 | < .001 | -44 | 22 | -6 |
| Posterior orbital |  |  |  |  |  | 4.67 | < .001 | -36 | 20 | -12 |
|  |  |  |  |  |  |  |  |  |  |  |
| Middle frontal | 6 | L | 0.009 | 161 | < .001 | 5.58 | < .001 | -34 | 6 | 48 |
| to precentral |  |  |  |  |  | 4.91 | < .001 | -42 | 8 | 50 |
|  |  |  |  |  |  | 4.82 | < .001 | -36 | 6 | 34 |
|  |  |  |  |  |  |  |  |  |  |  |
| Middle occipital | 17 | L | 0.013 | 148 | 0.001 | 5.55 | < .001 | -26 | -80 | 0 |
|  |  |  |  |  |  | 5.34 | < .001 | -22 | -80 | 10 |
|  |  |  |  |  |  |  |  |  |  |  |
| Parahippocampal to fusiform | 19 | L | 0.048 | 109 | 0.003 | 5.54 | < .001 | -26 | -40 | -8 |
|  |  |  |  |  |  | 4.71 | < .001 | -20 | -36 | -12 |
| Lingual |  |  |  |  |  | 4.55 | < .001 | -28 | -48 | -8 |
|  |  |  |  |  |  |  |  |  |  |  |
| Superior parietal | 7 | L | < .001 | 302 | < .001 | 5.1 | < .001 | -18 | -56 | 48 |
|  |  |  |  |  |  | 4.98 | < .001 | -22 | -52 | 28 |
|  |  |  |  |  |  | 4.97 | < .001 | -20 | -44 | 42 |
|  |  |  |  |  |  |  |  |  |  |  |
| ***Monetary won > Neutral won (Prosocial+)*** | | | | | | | | | | |
| Anterior cingulate | 10 | R | < .001 | 5119 | < .001 | 8.88 | < .001 | 8 | 32 | 6 |
| Inferior frontal (orbital) | 32 |  |  |  |  | 8.45 | < .001 | 44 | 34 | -8 |
| Orbital |  |  |  |  |  | 7.66 | < .001 | 34 | 38 | -2 |
|  |  |  |  |  |  |  |  |  |  |  |
| Inferior frontal (triangularis) | 13 | L | < .001 | 8305 | < .001 | 8.7 | < .001 | -46 | 22 | 2 |
| Thalamus |  |  |  |  |  | 8.13 | < .001 | -8 | -6 | 16 |
| to caudate |  | R |  |  |  | 8.07 | < .001 | 14 | 10 | 4 |
|  |  |  |  |  |  |  |  |  |  |  |
| Precentral to inferior frontal (opercularis) | 9 | R | 0.007 | 171 | < .001 | 7.19 | < .001 | 58 | 14 | 28 |
|  |  |  |  |  |  | 5.44 | < .001 | 52 | 12 | 38 |
|  |  |  |  |  |  | 4.45 | < .001 | 42 | 6 | 22 |
|  |  |  |  |  |  |  |  |  |  |  |
| Superior temporal | 40 | R | 0.02 | 140 | 0.001 | 6.99 | < .001 | 64 | -38 | 18 |
|  |  |  |  |  |  | 5.71 | < .001 | 60 | -42 | 12 |
|  |  |  |  |  |  | 5.18 | < .001 | 50 | -42 | 10 |
|  |  |  |  |  |  |  |  |  |  |  |
| Cerebellum |  | R | 0.001 | 235 | < .001 | 6.65 | < .001 | 10 | -42 | -30 |
| Brain stem |  | L |  |  |  | 5.54 | < .001 | -6 | -30 | -28 |
|  |  | R |  |  |  | 5.27 | < .001 | 10 | -50 | -42 |
|  |  |  |  |  |  |  |  |  |  |  |
| Precuneus | 7 | R | 0.015 | 149 | 0.001 | 6.64 | < .001 | 12 | -56 | 50 |
|  |  |  |  |  |  | 4.96 | < .001 | 6 | -42 | 50 |
|  |  |  |  |  |  | 4.6 | < .001 | 10 | -50 | 42 |
|  |  |  |  |  |  |  |  |  |  |  |
| Precuneus | 30/29 | L | 0.001 | 262 | < .001 | 5.83 | < .001 | -14 | -56 | 14 |
|  |  | R |  |  |  | 5.76 | < .001 | 2 | -54 | 16 |
|  |  | L |  |  |  | 5.06 | < .001 | -6 | -54 | 6 |
|  |  |  |  |  |  |  |  |  |  |  |
| ***Social won > Neutral won (Admiration-)*** | | | | | | | | | | |
| Precuneus | 23 | R | 0.016 | 134 | 0.001 | 5.58 | < .001 | 8 | -60 | 18 |
| Posterior cingulate |  |  |  |  |  | 5.17 | < .001 | 12 | -46 | 4 |
|  |  |  |  |  |  | 4.38 | < .001 | 6 | -54 | 8 |
|  |  |  |  |  |  |  |  |  |  |  |
| ***Social won > Neutral won (Prosocial+)*** | | | | | | | | | | |
| Precuneus | 7 | R | 0.026 | 121 | 0.001 | 7.09 | < .001 | 8 | -54 | 54 |
| Caudate |  |  | 0.022 | 126 | 0.001 | 6.57 | < .001 | 8 | 10 | -2 |
|  |  |  |  |  |  | 4.7 | < .001 | 6 | 22 | -18 |
| Anterior cingulate |  | L |  |  |  | 4.51 | < .001 | -2 | 26 | -10 |
|  |  |  |  |  |  |  |  |  |  |  |
| Anterior insula | 13 | L | < .001 | 707 | < .001 | 6.23 | < .001 | -32 | -6 | 12 |
| to posterior insula and putamen |  |  |  |  |  | 6.02 | < .001 | -30 | -24 | 16 |
|  |  |  |  |  |  | 5.66 | < .001 | -34 | -4 | 0 |
|  |  |  |  |  |  |  |  |  |  |  |
| Central operculum | 43 | L | 0.002 | 197 | < .001 | 6.03 | < .001 | -58 | -6 | 18 |
|  |  |  |  |  |  | 5.92 | < .001 | -56 | -8 | 10 |
|  |  |  |  |  |  | 5.13 | < .001 | -48 | -14 | 10 |
|  |  |  |  |  |  |  |  |  |  |  |
| Precentral gyrus | 6 | R | < .001 | 370 | < .001 | 5.92 | < .001 | 18 | -14 | 74 |
| Superior frontal |  |  |  |  |  | 5.82 | < .001 | 12 | -6 | 74 |
| Supplementary motor |  |  |  |  |  | 5.62 | < .001 | 4 | -4 | 66 |
|  |  |  |  |  |  |  |  |  |  |  |
| Thalamus (ventral lateral nucleus) |  | R | 0.001 | 234 | < .001 | 5.87 | < .001 | 14 | -10 | 2 |
|  |  |  |  |  |  | 5.64 | < .001 | 4 | -20 | 0 |
|  |  | L |  |  |  | 4.98 | < .001 | -6 | -16 | -2 |
|  |  |  |  |  |  |  |  |  |  |  |
| Precentral | 6 | R | 0.002 | 206 | < .001 | 5.74 | < .001 | 46 | -10 | 44 |
|  |  |  |  |  |  | 5.47 | < .001 | 36 | -12 | 38 |
|  |  |  |  |  |  | 4.87 | < .001 | 60 | -10 | 42 |
|  |  |  |  |  |  |  |  |  |  |  |
| Postcentral | 3/4 | R | 0.003 | 186 | < .001 | 5.71 | < .001 | 34 | -36 | 64 |
|  |  |  |  |  |  | 5.17 | < .001 | 30 | -34 | 56 |
| Superior parietal |  |  |  |  |  | 5.04 | < .001 | 22 | -40 | 64 |
|  |  |  |  |  |  |  |  |  |  |  |
| Central operculum to insula | 13 | R | 0.032 | 115 | 0.002 | 5.65 | < .001 | 46 | -6 | 18 |
|  |  |  |  |  |  | 5.25 | < .001 | 48 | -16 | 22 |
|  |  |  |  |  |  | 4.38 | < .001 | 50 | -12 | 12 |
|  |  |  |  |  |  |  |  |  |  |  |
| Precentral | 6 | R | 0.001 | 230 | < .001 | 5.49 | < .001 | 36 | -2 | 50 |
| Superior frontal |  |  |  |  |  | 5.42 | < .001 | 26 | -8 | 62 |
| Middle frontal |  |  |  |  |  | 4.88 | < .001 | 34 | 0 | 58 |
|  |  |  |  |  |  |  |  |  |  |  |
| Putamen |  | R | 0.038 | 110 | 0.002 | 5 | < .001 | 32 | 0 | -8 |
|  |  |  |  |  |  | 4.7 | < .001 | 26 | 6 | -14 |
|  |  |  |  |  |  |  |  |  |  |  |
| ***Social won > Neutral won (Prosocial-)*** | | | | | | | | | | |
| Superior temporal | 22 | L | 0.003 | 187 | < .001 | 6.87 | < .001 | -58 | -40 | 14 |
|  |  |  |  |  |  | 6.72 | < .001 | -56 | -34 | 8 |
|  |  |  |  |  |  |  |  |  |  |  |
| Middle occipital to cuneus | 18/30 | R | 0.028 | 119 | 0.001 | 5.94 | < .001 | 26 | -80 | 2 |
|  |  |  |  |  |  | 5.67 | < .001 | 32 | -74 | 2 |
|  |  |  |  |  |  | 5.09 | < .001 | 28 | -70 | 12 |
|  |  |  |  |  |  |  |  |  |  |  |
| Middle temporal | 19 | L | 0.032 | 115 | 0.002 | 5.22 | < .001 | -48 | -58 | 2 |
|  |  |  |  |  |  | 4.9 | < .001 | -38 | -52 | -4 |
|  |  |  |  |  |  | 4.12 | 0.001 | -36 | -42 | -6 |
|  |  |  |  |  |  |  |  |  |  |  |
| ***Monetary won > Social won (Prosocial+)*** | | | | | | | | | | |
| Middle frontal | 46 | R | < .001 | 322 | < .001 | 6.68 | < .001 | 26 | 42 | 18 |
|  |  |  |  |  |  | 5.49 | < .001 | 50 | 40 | 18 |
|  |  |  |  |  |  | 5.32 | < .001 | 32 | 42 | 8 |
|  |  |  |  |  |  |  |  |  |  |  |
| Middle frontal | 9 | L | 0.009 | 189 | 0.001 | 6.24 | < .001 | -28 | 38 | 18 |
|  |  |  |  |  |  | 5.13 | < .001 | -22 | 46 | 16 |
|  |  |  |  |  |  | 4.73 | < .001 | -18 | 44 | 24 |
|  |  |  |  |  |  |  |  |  |  |  |
| Anterior cingulate | 32 | R | 0.001 | 288 | < .001 | 5.95 | < .001 | 6 | 38 | 14 |
|  |  |  |  |  |  | 5.55 | < .001 | 12 | 34 | 10 |
|  |  |  |  |  |  | 5.36 | < .001 | 12 | 38 | -2 |
|  |  |  |  |  |  |  |  |  |  |  |
| Superior temporal | 22 | L | < .001 | 346 | < .001 | 5.74 | < .001 | -58 | -42 | 14 |
|  |  |  |  |  |  | 5.68 | < .001 | -52 | -34 | 6 |
|  |  |  |  |  |  | 4.3 | < .001 | -56 | -52 | 10 |
|  |  |  |  |  |  |  |  |  |  |  |
| ***Social won > Monetary won (Prosocial+)*** | | | | | | | | | | |
| Supplementary motor area | 6 | R | < .001 | 321 | < .001 | 5.85 | < .001 | 6 | -18 | 70 |
| Precentral |  |  |  |  |  | 4.63 | < .001 | 18 | -16 | 74 |
|  |  | L |  |  |  | 4.58 | < .001 | -14 | -16 | 72 |

*Note*: FWE – Family-wise error; MSIDT – Monetary and Social Intention Delay Task; SRQ – Social Reward Questionnaire
